# Insights into Polyprotein Processing and RNA-Protein Interactions in Foot-and-Mouth Disease Virus Genome Replication

**DOI:** 10.1128/jvi.00171-23

**Published:** 2023-05-08

**Authors:** Danielle M. Pierce, Connor Hayward, David J. Rowlands, Nicola J. Stonehouse, Morgan R. Herod

**Affiliations:** a School of Molecular and Cellular Biology, Faculty of Biological Sciences and Astbury Centre for Structural Molecular Biology, University of Leeds, Leeds, United Kingdom; Instituto de Biotecnologia/UNAM

**Keywords:** FMDV, picornavirus, cleavage, replication, replication complex, foot-and-mouth disease virus

## Abstract

Foot-and-mouth disease virus (FMDV) is a picornavirus, which infects cloven-hoofed animals to cause foot-and-mouth disease (FMD). The positive-sense RNA genome contains a single open reading frame, which is translated as a polyprotein that is cleaved by viral proteases to produce the viral structural and nonstructural proteins. Initial processing occurs at three main junctions to generate four primary precursors; L^pro^ and P1, P2, and P3 (also termed 1ABCD, 2BC, and 3AB_1,2,3_CD). The 2BC and 3AB_1,2,3_CD precursors undergo subsequent proteolysis to generate the proteins required for viral replication, including the enzymes 2C, 3C^pro^, and 3D^pol^. These precursors can be processed through both *cis* and *trans* (i.e., intra- and intermolecular proteolysis) pathways, which are thought to be important for controlling virus replication. Our previous studies suggested that a single residue in the 3B_3_-3C junction has an important role in controlling 3AB_1,2,3_CD processing. Here, we use *in vitro* based assays to show that a single amino acid substitution at the 3B_3_-3C boundary increases the rate of proteolysis to generate a novel 2C-containing precursor. Complementation assays showed that while this amino acid substitution enhanced production of some nonenzymatic nonstructural proteins, those with enzymatic functions were inhibited. Interestingly, replication could only be supported by complementation with mutations in *cis* acting RNA elements, providing genetic evidence for a functional interaction between replication enzymes and RNA elements.

**IMPORTANCE** Foot-and-mouth disease virus (FMDV) is responsible for foot-and-mouth disease (FMD), an important disease of farmed animals, which is endemic in many parts of the world and can results in major economic losses. Replication of the virus occurs within membrane-associated compartments in infected cells and requires highly coordinated processing events to produce an array of nonstructural proteins. These are initially produced as a polyprotein that undergoes proteolysis likely through both *cis* and *trans* alternative pathways (i.e., intra- and intermolecular proteolysis). The role of alternative processing pathways may help coordination of viral replication by providing temporal control of protein production and here we analyze the consequences of amino acid substitutions that change these pathways in FMDV. Our data suggest that correct processing is required to produce key enzymes for replication in an environment in which they can interact with essential viral RNA elements. These data further the understanding of RNA genome replication.

## INTRODUCTION

Small RNA viruses such as members of the *Picornaviridae* have limited genome size and therefore minimal coding capacity. These viruses have evolved several strategies to overcome this limitation, including the use of protein precursors that can perform different functions to the mature proteins. Furthermore, individual proteins and their precursors can also sometimes perform more than one function (1–4).

The *Picornaviridae* family includes several important human and animal pathogens, including but not limited to poliovirus (PV) and foot-and-mouth disease virus (FMDV). PV is responsible for the incapacitating (and potentially fatal) human disease poliomyelitis, while FMDV is the causative agent of the economically damaging foot-and-mouth disease, an acute vesicular disease of livestock, including cloven-hoofed ruminants and pigs. The FMDV genome contains one large open reading frame that encodes a ~250 kDa polyprotein (5). Initial processing of the FMDV polyprotein occurs at three positions to produce four primary products: L^pro^, the capsid precursor P1-2A and two nonstructural protein precursors 2BC (also termed P2) and 3AB_1,2,3_CD (also termed P3) (6, 7). L^pro^ is autocatalytically released from the N-terminal region of the polyprotein (6, 8). The P1-2A precursor is released from the polyprotein via a co-translational 2A-driven ribosome skipping mechanism (9) before the 2BC-3AB_1,2,3_CD polyprotein is processed by 3C^pro^. For FMDV, initial processing is believed to be predominantly at the 2C-3A junction, generating 2BC and 3AB_1,2,3_CD precursors. Further 3C^pro^-mediated proteolysis releases the final proteins via a succession of intermediate precursors (7, 10, 11). Processing of the 2BC precursor ultimately generates the 2B and 2C proteins, both of which have multiple roles in replication. The 3AB_1,2,3_CD precursor is composed of the transmembrane protein 3A, three 3B peptides (individually referred to as 3B_1_, 3B_2_, and 3B_3,_), the protease 3C^pro^, and the polymerase 3D^pol^ (12, 13).

Processing of the nonstructural polyprotein by 3C^pro^ is thought to occur through at least two separate pathways to generate mutually exclusive sets of precursors (14). For example, for FMDV, the 3AB_1,2,3_CD precursor is processed to generate the precursors 3AB_1,2,3_C and 3CD, which must be derived from alternative processing strategies. Likewise, for PV, the 3ABCD precursor (the equivalent of 3AB_1,2,3_CD in FMDV) can be processed to generate 3ABC and 3CD. Furthermore, it appears that this alternative processing may be temporally controlled and used to regulate virus replication. For example, previous studies with PV have demonstrated that later production of 3AB and 3CD can delay the initiation of viral RNA replication (15). For FMDV, reducing cleavage of 3CD inhibits replication by limiting the supply of 3D^pol^ (16). Processing through alternative pathways is likely to be driven (in part) through a switch between intramolecular versus intermolecular proteolysis (i.e., *cis* versus *trans* cleavage events). However, methods to differentiate between these *cis* versus *trans* cleavage events are challenging, and as a result, the mechanism(s) that controls this switch are not completely understood.

Like all positive-sense RNA viruses, picornavirus genome replication is associated with virus-induced cytoplasmic membranous structures, sometimes referred to as “replication complexes” or “replication organelles” (17). In these assemblies, multiple new viral positive-strand RNAs are synthesized via a complementary negative-sense template. For FMDV, the full composition of these assemblies is unknown, but they are likely composed of multiple viral and cellular factors, including the nonstructural proteins 3B, 3D^pol^, and 3CD. Some of the viral nonstructural proteins and precursors associate with RNA elements located in the 5′ and 3′ untranslated regions (UTRs) that flank the open reading frame (5). The 5′ UTR of FMDV is uncharacteristically long for a picornavirus and contains several distinct structural elements, including an internal ribosome entry site (IRES), a *cis*-acting replicative element (*cre*), a large stem-loop (termed the S-fragment) and a tandem series of pseudoknots (18–21). The IRES has been well studied and is used to initiate protein translation in a cap-independent manner (19, 20). The *cre* is essential for replication and acts as the template for uridylation of 3B (also known as VPg) to generate the replicative primer, VPg-pUpU (22–27). The role of the S-fragment in FMDV replication is less well understood but may be involved in both replication and modulating the innate immune response (28). In other picornaviruses, such as PV, an RNA structure termed the cloverleaf (or oriL) is located at the 5′ terminus of the genome at the site occupied by the S-fragment stem-loop in FMDV (29). This interacts with the precursor protein 3CD as well as host proteins and is involved in initiating negative-strand RNA synthesis (30–35). Furthermore, for PV, other precursor proteins have also been implicated in 5′ UTR interactions, including 3AB (the equivalent to 3AB_1,2,3_ in FMDV), 3BCD, and 3ABCD, and have been suggested to be important for controlling replication (15, 31, 36, 37). The role of precursors in FMDV replication is less well established.

In a previous study, we used an FMDV replicon system based on serotype O1K, where replication is monitored by fluorescent protein (e.g., GFP/RFP) expression over time, to investigate FMDV replication by mutation of the 3B proteins (16). We reported a series of amino acid substitutions that increased the efficiency of processing at the 3B_3_-3C junction but inhibited replication by abrogating the release of free 3D^pol^. Simultaneously, we observed that these substitutions caused an overall shift in 3AB_1,2,3_CD processing and channeled precursor synthesis mainly down one pathway generating 3AB_1,2,3_ and 3CD precursors. This series of mutations has enabled us to separate alternative cleavage pathways and study the function of different precursor sets. Here, we investigated the mechanism by which these substitutions result in increased processing between 3B_3_ and 3C. Our data suggest that a single amino acid substitution increases sensitivity to *trans*-mediated proteolysis at this boundary. Furthermore, when placed into the context of a full-length polyprotein, this single substitution resulted in accumulation of a novel precursor. Interestingly, it also prevented reciprocal complementation of replicons in *trans*, which we demonstrate is due to a deficiency in the functions of essential viral enzymes.

## RESULTS

### A single amino acid substitution in 3B_3_ prevents replicon replication.

In a previous study, we reported that amino acid substitutions within 3B_3_, at the boundary with 3C^pro^, dramatically changed processing of the FMDV 3AB_1,2,3_CD polyprotein and prevented release of active 3D^pol^. However, through blind passage, a compensatory mutation was selected which restored replication and wild-type (WT) 3AB_1,2,3_CD processing. This was identified as a reversion of a lysine at the P2 residue of the 3B_3_-3C junction to the WT threonine (16). These data suggested that the amino acid in the P2 position of the 3B_3_-3C junction alone can be a major determinant of altered polyprotein processing.

**FIG 1:**
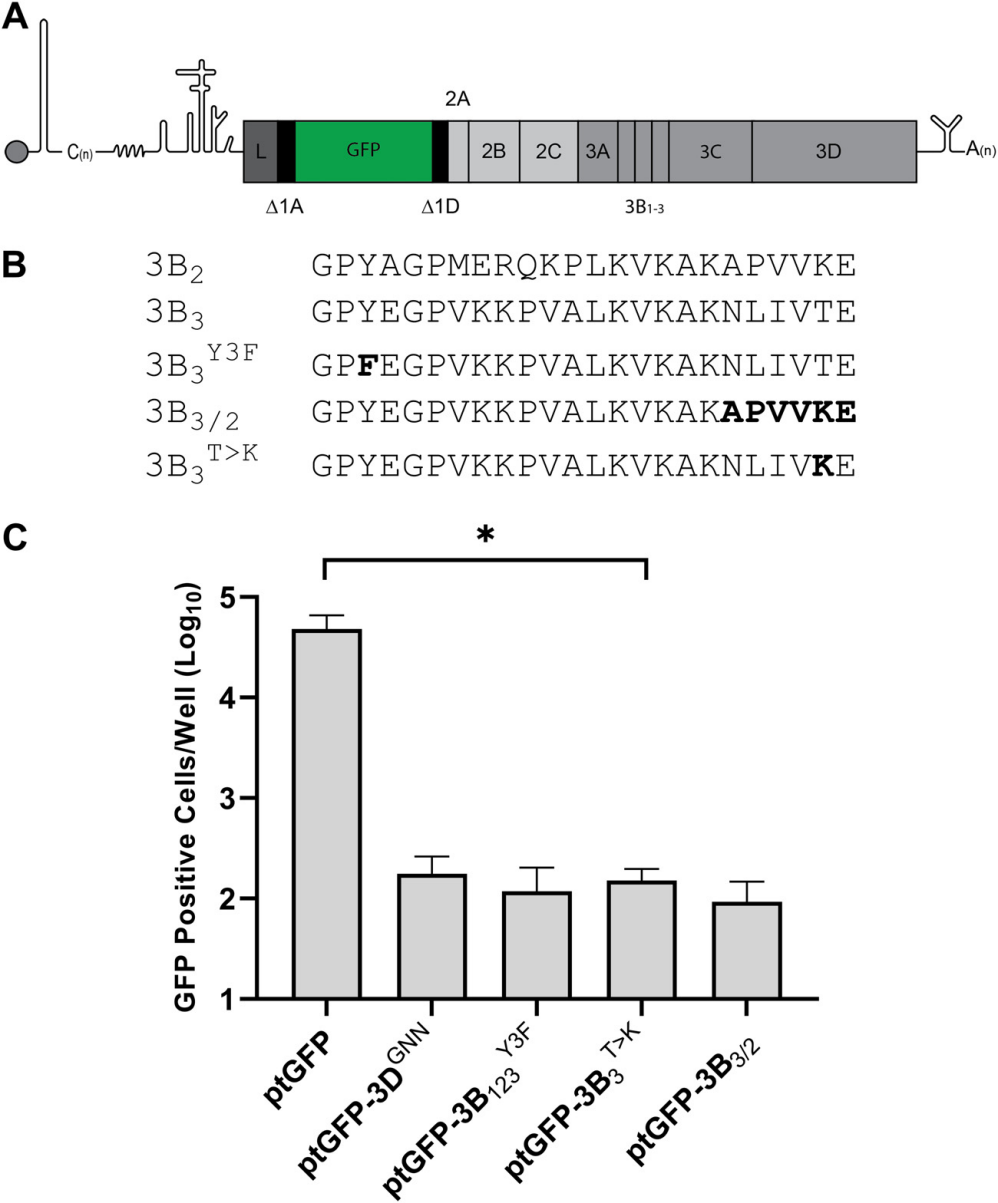
A single amino acid substitution at the 3B_3_-3C junction prevents FMDV replicon replication. (A) Schematic diagram of the FMDV replicon. (B) Sequence alignments of the 3B cleavage junctions with the 3B_1,2,3_^Y3F^, 3B_3/2_, and 3B_3_^T>K^ mutants. (C) Replication of replicons containing 3B_3_ mutations as well as the WT ptGFP replicon (ptGFP) and replication-defective controls containing inactivating mutations in 3D^pol^ (3D^GNN^), or 3B proteins (3B_1,2,3_^Y3F^). GFP expression was monitored hourly for 24 h. The graph shows GFP positive cells per well at 8 h posttransfection when replication is maximal. Significance compared to WT control (*n* = 3 ± SEM; * = *P* ≤ 0.05).

Before investigating the mechanism by which substitutions at this junction increased processing, we first sought to establish that this residue alone was sufficient to change FMDV polyprotein processing and prevent replicon replication. To this end, we generated an FMDV replicon with a threonine to lysine substitution at the P2 residue of the 3B_3_-3C cleavage junction (Fig. 1A and B). In this replicon (termed ptGFP-3B_3_^T>K^) the reporter protein ptGFP replaced the structural proteins, allowing ptGFP expression to be used as an indicator of replicon replication (Fig. 1A). This replicon RNA was transfected into BHK-21 cells alongside the previously published mutant replicons ptGFP-3B_1,2,3_^Y3F^ (contains inactivating point mutations to the triptych of 3B genes) and ptGFP-3B_3/2_ (the six C-terminal residues of 3B_3_ replaced by those of 3B_2_) (Fig. 1B). A WT ptGFP expressing replicon (ptGFP) and a replicon containing an inactivating double point mutation in 3D^pol^ (ptGFP-3D^GNN^) were included as controls. The latter replicon serves as a negative control for ptGFP production from translation of the input transfected RNA, as we have previously described (38). RNAs from these replicons were transfected into BHK-21 cells and replication monitored by ptGFP fluorescence using an Incucyte real-time imaging system (Fig. 1C).

As anticipated, the WT ptGFP replicon produced ptGFP >100-fold greater than the ptGFP-3D^GNN^ control replicon, as previously reported (39). In comparison, the ptGFP-3B_3_^T>K^ replicon showed GFP expression equivalent to the replication-defective ptGFP-3D^GNN^ control. These data were in agreement with those obtained with the ptGFP-3B_3/2_ and ptGFP-3B_1,2,3_^Y3F^ replication-defective replicons we previously reported (16). Thus, the 3B_3_^T>K^ substitution alone is sufficient to prevent replicon replication.

### A single amino acid substitution at the 3B_3_-3C boundary increases the rate of proteolysis.

**FIG 2:**
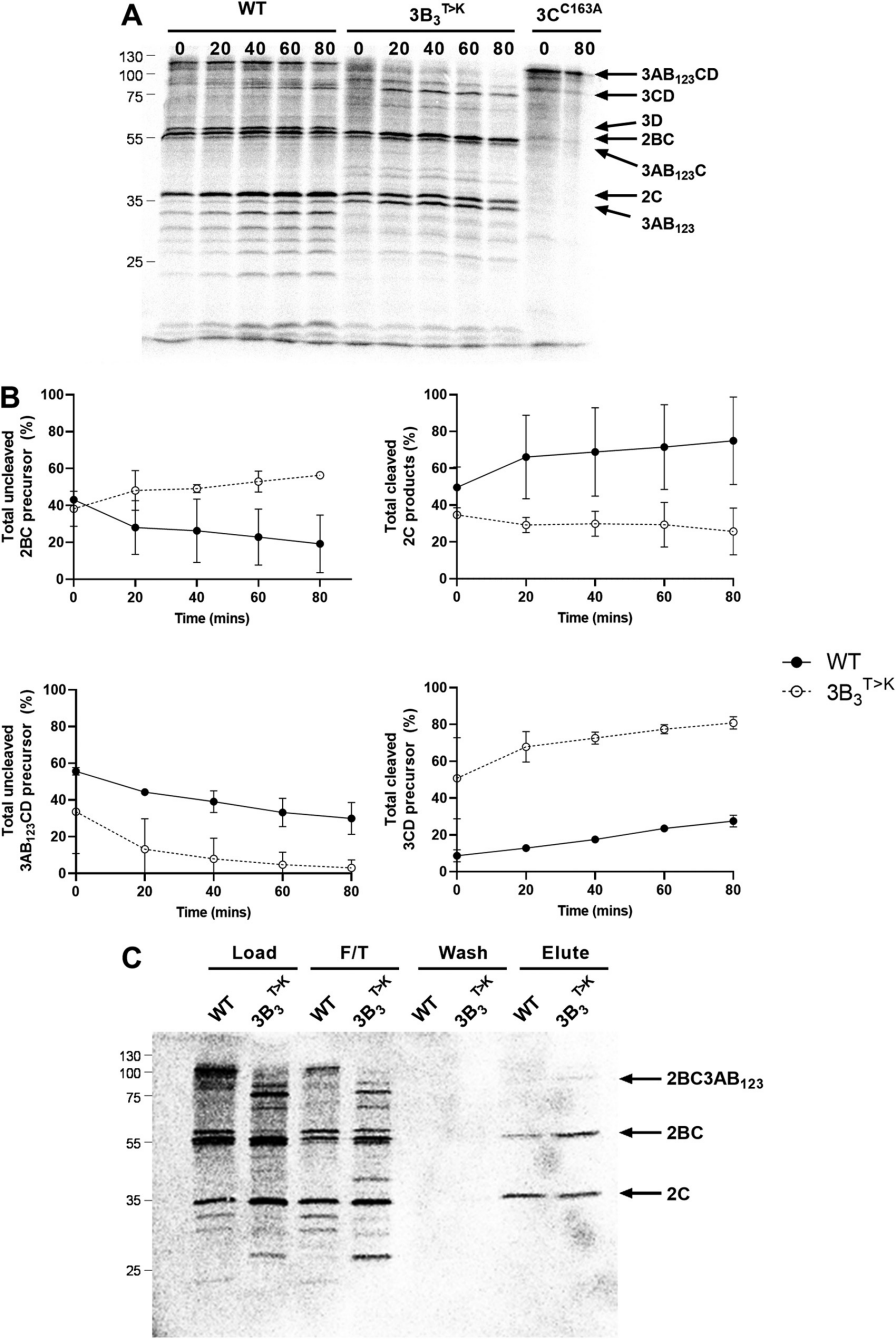
A single amino acid substitution at the 3B_3_-3C boundary increases the rate of proteolysis and drives the production of novel precursors. (A) Plasmids expressing the WT or mutant 3B_3_^T>K^ FMDV polyprotein precursors were used to prime [^35^S] labeled pulse-chase *in vitro* coupled transcription/translation reactions. At regular intervals, samples were taken and stopped by the addition of 2× Laemmli buffer. Proteins were separated by SDS-PAGE and visualized by autoradiography. The identity of some FMDV proteins is shown. (B) The percentage of protein or protein precursor was quantified as a total percentage of 2C or 3D^pol^ containing products, as appropriate (*n* = 2 ± SD). (C) Duplicate reactions were incubated for 90 min before immunoprecipitation of 2C containing precursors with anti-2C antibodies. The pre- and post precipitation samples were separated by SDS-PAGE and visualized by autoradiography. Arrows show the identity of 2C containing proteins, based on predicted molecular weights. F/T refers to the flow through samples.

We previously demonstrated that the ptGFP-3B_3/2_ mutation inhibited replication and changed processing of the 3AB_1,2,3_CD polyprotein. To confirm that the 3B_3_^T>K^ substitution alone was sufficient to induce the same changes, we employed the previously described *in vitro* coupled transcription/translation assay (16). T7 expression constructs were generated to express either the WT FMDV 2BC-3AB_1,2,3_CD polyprotein or a polyprotein containing the 3B_3_^T>K^ amino acid substitution. For simplicity, in these experiments L^pro^ and GFP were not included in the polyprotein. The polyprotein used in these experiments included 2BC as well as the 3AB_1,2,3_CD region to determine changes to the entire NS polyprotein. These experiments also included a control polyprotein containing an inactivating mutation in 3C^pro^ (3C^C163A^) predicted to prevent its proteolytic activity (40). Processing was investigated by [^35^S] methionine/cysteine pulse/chase labeling in *in vitro* coupled transcription/translation reactions, harvesting samples at regular time points and analyzing protein products by SDS-PAGE (Fig. 2A and B).

For the WT construct, the full-length 3AB_1,2,3_CD precursor was detected at early time points, and was steadily processed over time primarily into 3AB_1,2,3_C and 3D^pol^, with a small amount of 3CD derived from an alternative processing pathway. At the later time points (40 and 60 min), 3AB_1,2,3_ was also detected. Both 2B and 2C were present, in addition to a limited amount of the precursor 2BC at earlier time points. Compared to WT, the construct containing the 3B_3_^T>K^ substitution resulted in greater amounts of the 3CD and 3AB_1,2,3_ precursors and less of the 3AB_1,2,3_CD precursor. There were also increased levels of the 2BC precursor in addition to a high molecular weight precursor, possibly 2BC3AB_1,2,3_, which was detected at early time points and gradually decreased over time. These data extended our previous observations demonstrating that the 3B_3_^T>K^ substitution alone is sufficient to accelerate proteolysis at the 3B_3_-3C junction and so increases the relative amounts of 2BC, 3CD, and 3AB_1,2,3_.

### Increasing polyprotein proteolysis generates a novel 2BC containing precursor.

The previous *in vitro* translation experiments suggested that the 3B_3_^T>K^ substitution increased the production of the 2BC precursor in addition to a larger molecular weight precursor not observed in the WT control. To confirm the identity of the 2C-containing precursors, the *in vitro* translation samples were immunoprecipitated using an anti-2C antibody. T7 constructs expressing the WT polyprotein or polyprotein containing the 3B_3_^T>K^ substitution were used in *in vitro* coupled transcription/translation reactions with [^35^S] methionine/cysteine pulse/chase labeling. Samples were taken after 90 min and immunoprecipitation performed on half of the sample. Both pre- and post-immunoprecipitation protein samples were analyzed by SDS-PAGE (Fig. 2C).

In comparison to WT, a smaller proportion of mature 2C (but more unprocessed 2BC precursor) were immunoprecipitated with an anti-2C antibody following expression from the polyprotein containing the 3B_3_^T>K^ substitution. Furthermore, the additional higher molecular weight band which was only present following expression of the 3B_3_^T>K^ precursor was also immunoprecipitated with the anti-2C antibody. Based on these observations and the estimated molecular weight, this product is mostly likely a 2BC3AB_1,2,3_ precursor. These results agree with previous data and suggest that the 3B_3_^T>K^ substitution preferentially increases the rate of proteolysis at the 3B_3_-3C junction, compared to the 2C-3A junction, resulting in the accumulation of 2BC3AB_1,2,3_, which is not normally detected.

### The 3B_3_^T>K^ substitution stimulates *trans*-mediated processing.

**FIG 3:**
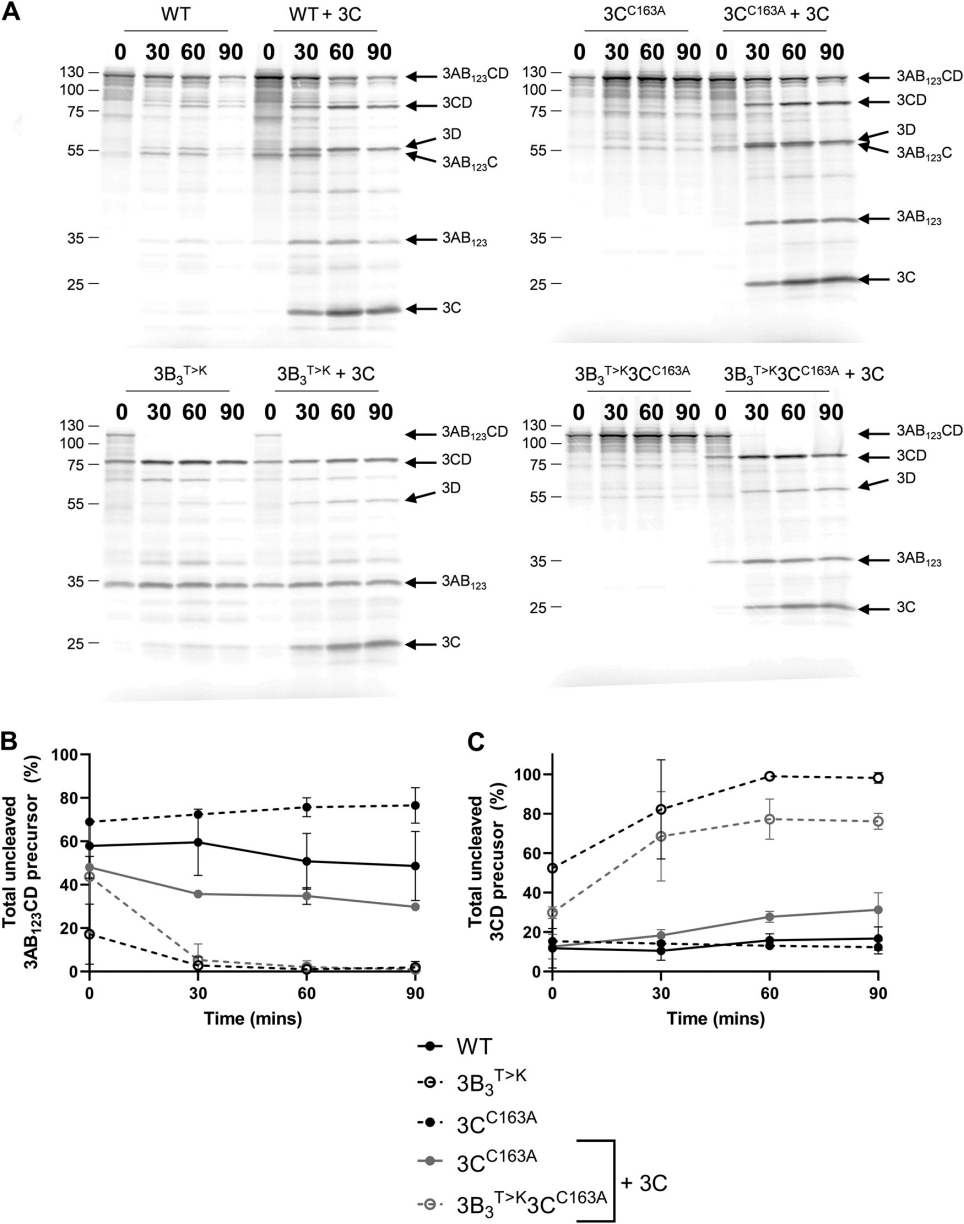
The 3B_3_^T>K^ amino acid substitution drives *trans*-mediated precursor proteolysis. Plasmids expressing proteolytically inactive polyproteins with or without the 3B_3_^T>K^ substitution (termed 3B_3_^T>K^, 3C^C163A^, and 3C^C163A^, respectively) were used to prime coupled [^35^S] labeled transcription/translation assays, followed by unlabeled amino acid chase. To a duplicate set of reactions 10 μM purified 3C^pro^ was added (+3C) immediately after chase. As controls, reactions were set up alongside the WT or 3B_3_^T>K^ polyproteins without an inactivated 3C^pro^. At regular intervals, samples were taken and reactions stopped by the addition of 2× Laemmli buffer. (A) Proteins were separated by SDS-PAGE and visualized by autoradiography. The relative proportion of uncleaved 3AB_1,2,3_CD (B) or 3CD (C) was quantified as a percentage of 3D^pol^ containing products (*n* = 2 ± SD).

We speculated that the most likely mechanism by which the order of polyprotein processing was altered was that the substitution generated a boundary sequence that was preferentially recognized in *trans* by 3C^pro^ and/or a 3C^pro^-containing precursor. To explore the latter possibility, we adapted our *in vitro* assay to investigate *trans*-mediated cleavage. For simplicity, we adapted both the WT 3AB_1,2,3_CD precursor or precursor containing the 3B_3_^T>K^ substitution to also contain the inactivating mutation in 3C^pro^ (3C^C163A^) to prevent self-proteolysis (40). These precursor substrates (termed 3C^C163A^ and 3B_3_^T>K^-3C^C163A^, respectively) were translated *in vitro* with [^35^S] methionine/cysteine before adding excess unlabeled methionine/cysteine and purified active 3C^pro^ to a duplicate set of reactions (plus 3C^pro^). Samples were harvested at regular time points and processing of the [^35^S] labeled precursor was analyzed by SDS-PAGE (Fig. 3). As controls, the experiment was conducted with the WT and 3B_3_^T>K^ constructs that did not contain the 3C^C163A^ point mutation (Fig. 3). To aid interpretation, the relative amounts of 3AB_1,2,3_CD and 3CD products were quantified by phosphorimaging (Fig. 3).

Both WT and 3B_3_^T>K^ precursors carrying the 3C^C163A^ mutation produced only uncleaved full-length 3AB_1,2,3_CD in the absence of 3C^pro^ provided in *trans*. The addition of 3C^pro^ resulted in the production of smaller proteins due to *trans*-mediated proteolysis of 3AB_1,2,3_CD. For the WT precursor, these were predominantly 3AB_1,2,3_, 3CD, and 3D^pol^, in addition to a cluster of 3B_1,2,3_CD-derived products, including some 3CD, indicative of alternative cleavage pathways. In comparison, the precursor containing the 3B_3_^T>K^ substitution was processed to 3AB_1,2,3_ and a greater proportion of 3CD compared to WT over the duration of the experiment, as observed previously with the active precursor molecule (Fig. 2A) (16).

**FIG 4:**
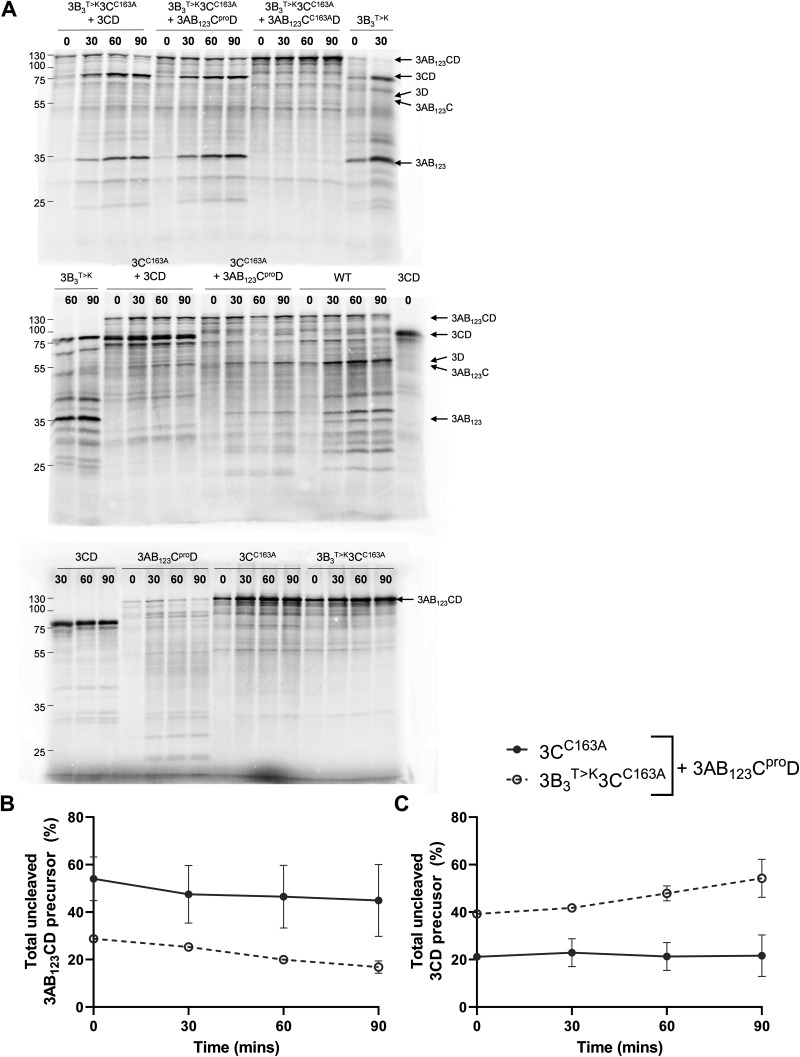
The 3B_3_^T>K^ amino acid substitution drives *trans*-mediated precursor proteolysis. Plasmids expressing proteolytically inactive versions of WT or 3B_3_^T>K^ polyproteins (3C^C163A^ and 3B_3_^T>K^3C^C163A^, respectively) were used to prime coupled [^35^S] labeled transcription/translation assays in the presence of 3AB_1,2,3_C^pro^D, a proteolytically active precursor with all cleavage boundaries mutated to prevent self-proteolysis (+3AB_123_C^pro^D). At regular intervals, samples were taken and reactions stopped by the addition of 2× Laemmli buffer. (A) Proteins were separated by SDS-PAGE and visualized by autoradiography. The relative proportion of uncleaved 3AB_1,2,3_CD (B) or 3CD (C) was quantified as a percentage of 3D^pol^ containing products (*n* = 2 ± SD).

To investigate whether the 3B_3_^T>K^ precursor was also sensitive to cleavage by 3C^pro^ when this is present as part of a larger precursor molecule, we generated a 3AB_1,2,3_CD expression construct in which all the cleavage boundaries had been mutated. Thus, the protease activity of this precursor was retained but only in the context of a full-length 3AB_1,2,3_CD polyprotein. Our *trans*-cleavage assay was repeated using this new construct, termed 3AB_1,2,3_C^pro^D, in place of the purified 3C^pro^ enzyme used above (Fig. 4).

As before, the 3C^C163A^ mutation prevented self-proteolysis when present within the WT precursor or the precursor containing the 3B_3_^T>K^ substitution, as expected. Addition of the proteolytically active 3AB_1,2,3_C^pro^D construct resulted in processing of the proteolytically inactive 3AB_1,2,3_CD precursor bearing the 3B_3_^T>K^ substitution to generate 3AB_1,2,3_ and 3CD. This pattern of processing was similar to that observed following addition of active 3C^pro^, as observed above (Fig. 3). This contrasts with the WT proteolytically inactive 3AB_1,2,3_CD precursor, which was not significantly processed in *trans* by 3AB_1,2,3_C^pro^D. Taken together, these data suggest that the 3B_3_^T>K^ substitution at the 3B_3_-3C junction generates a cleavage boundary that is preferentially recognized by 3C^pro^ (even when delivered as part of a larger precursor). Thus, driving rapid *trans*-mediated proteolysis at this junction results in overproduction of a specific set of viral precursor proteins.

### Increasing the rate of 3B_3_-3C cleavage prevents production of *trans*-functional 2C and 3D^pol^ but not 3B.

**FIG 5:**
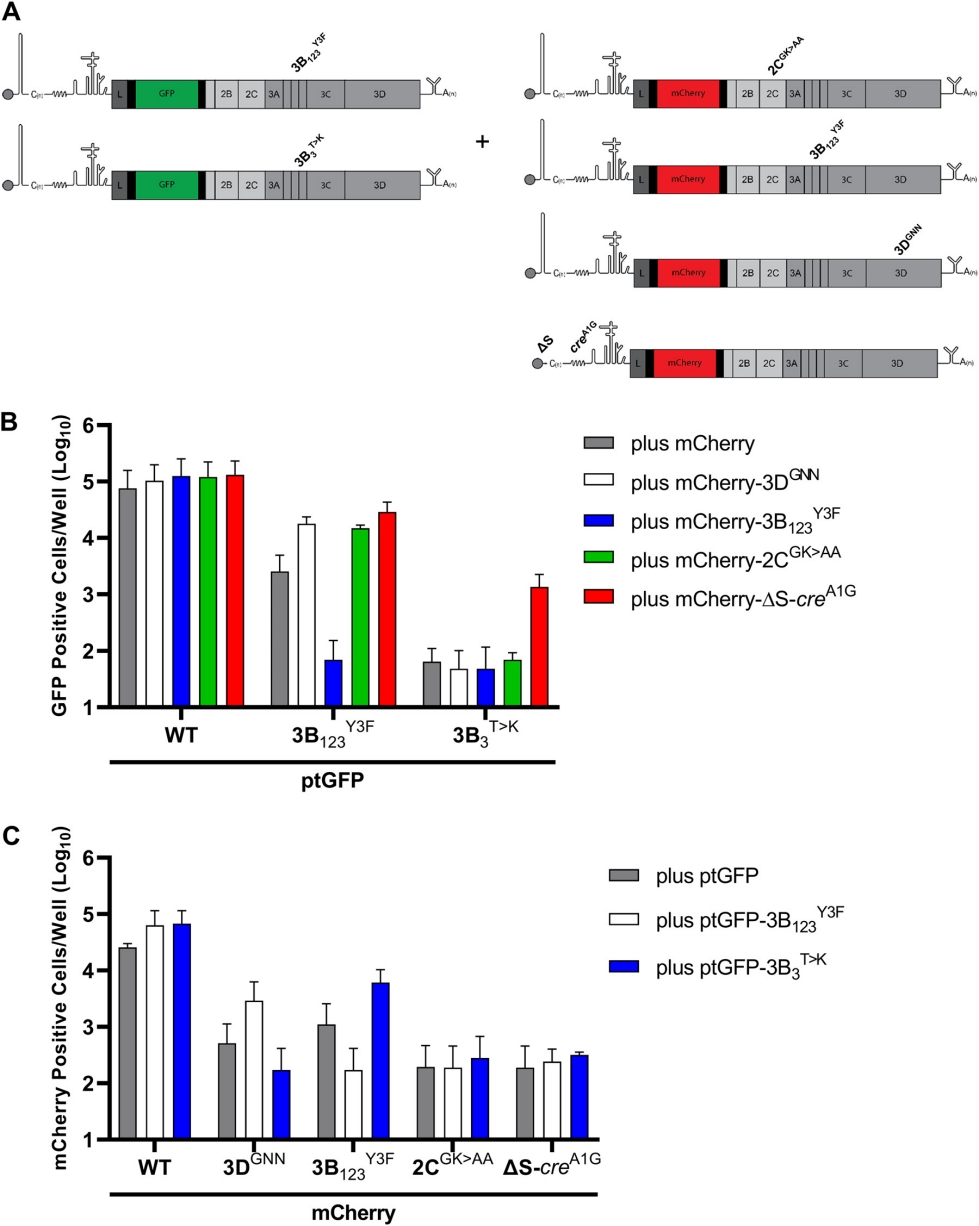
The 3B_3_^T>K^ substitution prevents complementation of 2C mutants in *trans*. (A) Schematic of the *trans*-complementation experiment which involved co-transfecting BHK-21 cells with mCherry replicons containing replication-defective 2C or 3B mutations together with a WT ptGFP, ptGFP-3B_3_^T>K^ or ptGFP-3D^GNN^ replicon. Fluorescent protein expression was monitored hourly for 24 h. The data show (B) ptGFP positive cells per well or (C) mCherry positive cells per well at 8 h posttransfection (*n* = 2 ± SD).

The *in vitro* polyprotein processing data above implies that the 3B_3_^T>K^ substitution increases the rate of proteolysis at the 3B_3_-3C junction by stimulating *trans*-mediated proteolysis. In doing so, it drives the formation of 2BC, 3AB_1,2,3_, and 3CD precursors to the detriment of other products such as the enzymes 2C and 3D^pol^. In our previous studies, we used *trans*-complementation assays to investigate protein function. These assays involve the co-transfection of two replication defective replicon constructs that express different fluorescent reporter genes, allowing replication to be differentially monitored. Co-transfection of the two replicons allows exchange of viral nonstructural proteins within replication complexes to permit replication of one (or both) of the input genomes (39) (Fig. 5A). Here, we used this approach to investigate whether stimulating *trans*-mediated proteolysis at the 3B_3_-3C junction prevented the production of functional 2C. To do this, inactivating substitutions introduced into 2C were investigated to determine if these could be compensated by a replicon harboring a 3B_3_^T>K^ substitution. This possibility would indicate that changing the temporal order of polyprotein processing does not prevent 2C function.

To this end, replication-defective mCherry constructs were generated which contained inactivating substitutions at catalytic 2C residues (termed, mCherry-2C^GK>AA^) (4). This construct was co-transfected with the ptGFP-3B_3_^T>K^ replicon (as used in Fig. 1C), or a WT ptGFP replicon control. As a positive control, co-transfections were also performed with an mCherry-3B_1,2,3_^Y3F^ replicon (which contains inactivating point mutations to the triptych of 3B genes), which we have shown can be complemented in *trans* (16). Co-transfections were also performed with WT mCherry or ptGFP replicons to eliminate the possibility of any dominant negative effects and yeast tRNA to act as a negative control for no complementation (Fig. 5A). Replication was monitored by both ptGFP and mCherry expression and the number of fluorescent positive cells quantified at 8 h posttransfection, as documented previously (39). For brevity, the key data sets and controls are shown (Fig. 5B and C) with the complete data set shown in Fig. S1.

Replication of the WT mCherry or ptGFP replicon did not significantly change upon co-transfection with any of the RNAs tested, suggestive of no dominant negative effects. Replication of the mCherry-3B_1,2,3_^Y3F^ replicon was significantly enhanced by the ptGFP-3B_3_^T>K^ construct, as anticipated. The mCherry-2C^GK>AA^ replicon was not recovered by any of the helper replicons, suggesting the functions of 2C cannot be provided in *trans* (Fig. 5C). We also noticed that no functional complementation was provided to the ptGFP-3B3^T>K^ replicon by co-transfection with mCherry-3B_1,2,3_^Y3F^. To investigate this further, we extended our complementation experiments to include mCherry replicons containing mutations to *cis*-acting RNA replication elements such as the S-fragment or *cre*. The rationale here was that previous studies have demonstrated that deleting *cis*-acting replication elements can improve or allow recovery of replicons in *trans* (39), presumably by increasing the free pool of proteins which would otherwise be sequestered by *cis* interactions. When the ptGFP-3B_3_^T>K^ replicon was co-transfected with a mCherry-ΔS or mCherry-*cre*^A1G^ replicon, we observed a significance increase in ptGFP expression, indicating that inactivation of these *cis*-acting replication elements permitted complementation of the ptGFP-3B_3_^T>K^ replicon in *trans* (Fig. 5B). Together, these data suggest that the ptGFP-3B_3_^T>K^ replicon can supply material in *trans* to recover replicons defective in 3B but not 2C or 3D^pol^ and can only receive complementation in *trans* from replicons with inactivating mutations or deletions to *cis*-acting RNA elements.

### Complementation of replicons with the 3B_3_^T>K^ substitution reveals a requirement for the nonstructural polyprotein.

**FIG 6:**
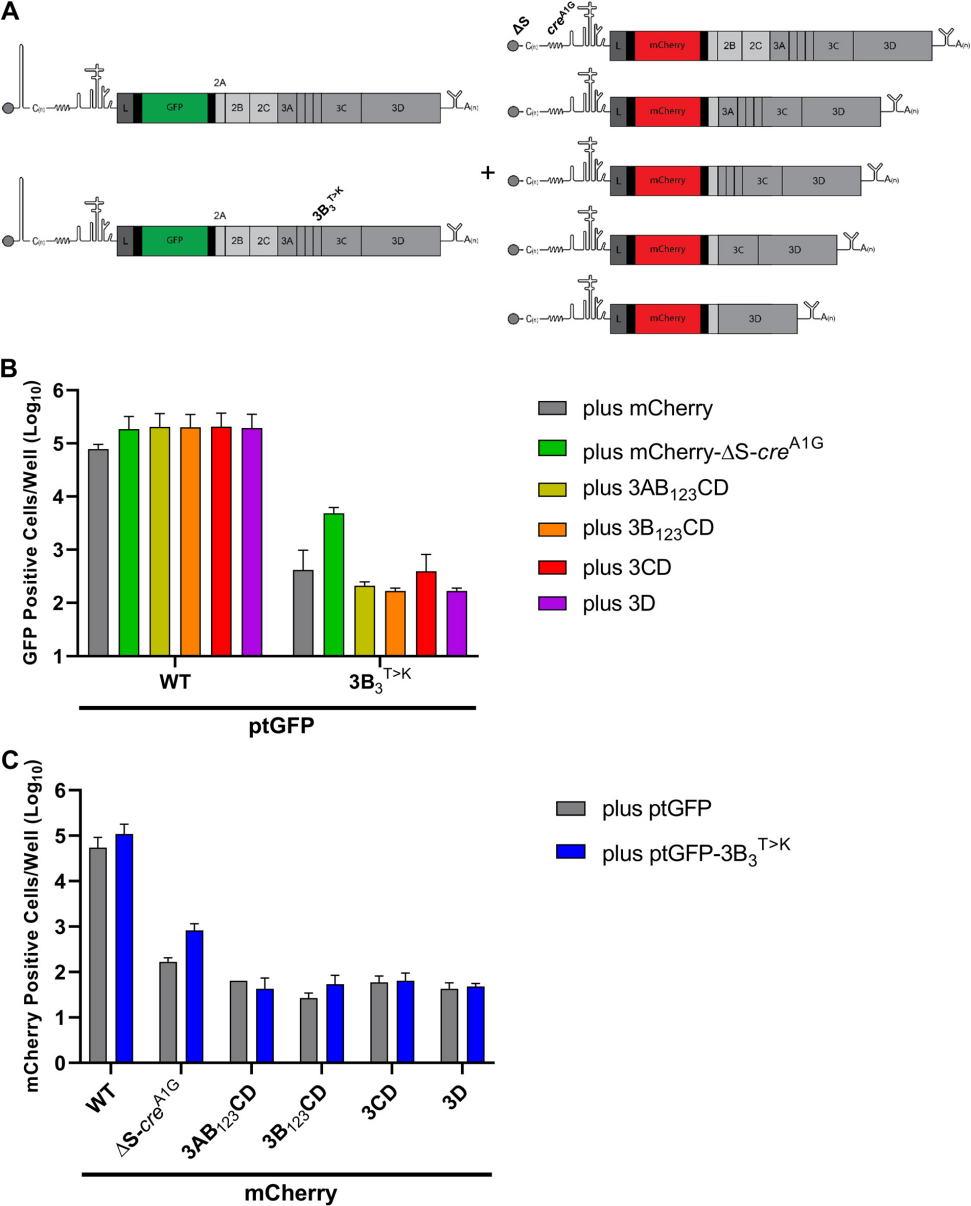
S-fragment deletions allow *trans*-complementation of *cis*-acting replication components. (A) Schematic of the trans-complementation experiment which involved co-transfecting BHK-21 cells with mCherry replicons containing S-fragment deletions together with a WT ptGFP, ptGFP-3B_3_^T>K^ or ptGFP-3D^GNN^ replicon. Fluorescent protein expression was monitored hourly for 24 h. The data show (B) ptGFP positive cells per well or (C) mCherry positive cells per well at 8 h posttransfection (*n* = 2 ± SD).

Having shown that deletion of *cis*-acting RNA elements allowed complementation of the ptGFP-3B_3_^T>K^ replicon, we took advantage of this system to identify which protein component was required for complementation. To this end, we generated a new panel of mCherry replicons in which both the S-fragment and *cre* were inactivated but which expressed just a subset of the polyprotein, namely, 3AB_1,2,3_CD, 3B_1,2,3_CD, 3CD, or 3D^pol^ (Fig. 6A). With these constructs, we were able to probe whether the ptGFP-3B_3_^T>K^ replicon is missing the ability to generate one of these protein components to initiate replication. This new panel of replicons was used in complementation assays with the same controls as described above, with replication monitored by both ptGFP and mCherry expression, and the number of fluorescent positive cells quantified at 8 h posttransfection. Again, for clarity, the key data sets and controls are shown (Fig. 6B and C) with the complete data set shown in Fig. S2.

As described above, we found that replicons lacking a functional S-fragment and/or *cre* were able to significantly increase the replication of the ptGFP-3B_3_^T>K^ replicon, with ptGFP expression increasing >10-fold compared to the controls. In contrast, no complementation of the ptGFP-3B_3_^T>K^ replicon was observed when co-transfected with RNAs expressing just 3AB_1,2,3_CD, 3B_1,2,3_CD, 3CD, or 3D^pol^. Hence, it would appear that 3AB_1,2,3_CD is not sufficient to support replication of a 3B_3_^T>K^ replicon and provision of 2BC containing proteins is also required.

### The 3B_3_^T>K^ substitution does not prevent RNA-protein interactions.

A key observation from our *trans*-complementation work is that the 3B_3_^T>K^ substitution prevents complementation of this nonfunctional replicon unless the *cis*-acting S-fragment or *cre* elements are deleted from the helper RNA. A possible explanation is that the altered cleavage pattern induced by the 3B_3_^T>K^ substitution prevents replication components (e.g., the 2C helicase or 3D^pol^) from interacting with the template RNA, thus preventing its replication.

**FIG 7:**
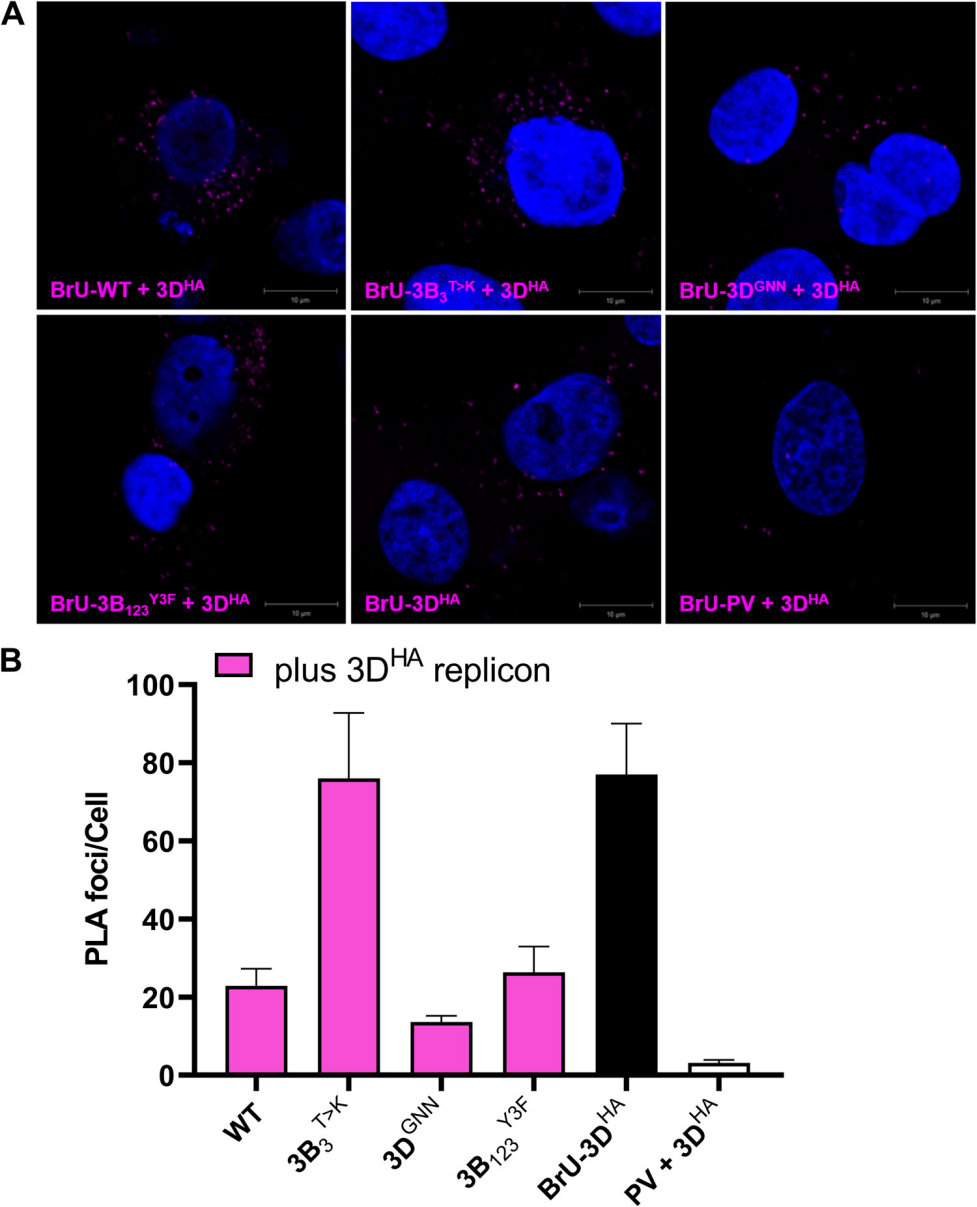
Detection of FMDV RNA-protein complexes by PLA. (A) BHK-21 cells were co-transfected with BrU-labeled ptGFP replicon RNA together with 3D^HA^-labeled replicon RNA. At 4 h posttransfection cells were fixed and 3D^HA^-BrU RNA complexes detected by proximity ligation assay (PLA) using anti-HA and anti-BrU primary antibodies together with PLA-labeled secondary antibodies. The *in-situ* PLA signal is detected as foci in the cell cytoplasm (pseudo-colored magenta). Cell nuclei were stained with DAPI (pseudo-colored blue). Images were captured on a Zeiss LSM-880 confocal microscope (bar 10 μm). (B) The mean number of individual PLA foci per cell were quantified from an average of at least 20 GFP positive cells per condition spread across two biological replicate experiments (*n* = 2 ± SD).

To investigate this possibility, we adapted a proximity ligation assay (PLA) to study interactions between replicon RNA and components of the replication complex. First, ptGFP-3B_3_^T>K^ replicon RNA (or ptGFP-3D^GNN^ and ptGFP-3B_1,2,3_^Y3F^ controls) was *in vitro* transcribed in the presence of BrUTP to generate BrU-labeled replicon RNA. Alongside, a WT ptGFP was transcribed with BrUTP and used to confirm that BrU labeling had no significant inhibitory effect on replicon replication (data not shown). The ptGFP-3B_3_^T>K^ BrU replicon was co-transfected into BHK-21 cells seeded on coverslips together with a WT replicon in which 3D^pol^ had been labeled with a HA epitope (termed 3D^HA^). HA epitopes are derived from the human influenza virus hemagglutinin (HA) protein and we have previously demonstrated that tagging 3D^pol^ with HA in this manner does not affect replication (39). The coverslips were fixed and the interaction between the BrU-3B_3_^T>K^ RNA and 3D^HA^ probed by PLA using anti-BrU and anti-HA antibodies. This allows RNA-protein interactions between the ptGFP-BrU-3B_3_^T>K^ RNA and 3D^HA^ protein to be measured *in situ*. A positive PLA signal indicates that the mutant replicon RNA can associate with enzymes of the replication complex provided in *trans*. No signal indicates a lack of association (Fig. 7A). The number of PLA foci per GFP positive cell was quantified from at least 20 cells per sample (Fig. 7B).

Co-transfection of the WT ptGFP BrU labeled replicon with the WT 3D^HA^ replicon generated PLA signals that were easily detectable, indicative of interactions between the ptGFP RNA and 3D^HA^ protein as predicted from mixing of replication complex components. In contrast, little or no PLA signal was detected when a WT BrU labeled poliovirus replicon (BrU-PV) was co-transfected with the 3D^HA^ helper replicon, with a ~40-fold decrease in the number of PLA foci/cell compared to the positive control. This suggests the PLA signal is generated through specific mixing of replication complexes. Co-transfection of the ptGFP-3B_3_^T>K^, ptGFP-3D^GNN^, or ptGFP-3B_1,2,3_^Y3F^ BrU labeled replicon RNAs with the WT 3D^HA^ replicon also generated PLA signals that were easily detectable, with ~40-, ~10-, and ~15-fold increase in PLA foci/cell compared to the BrU-PV control. This would suggest the 3B_3_^T>K^ substitution does not prevent recruitment of its cognate RNA with a functional RNA polymerase.

## DISCUSSION

All well-studied positive-sense RNA viruses produce polyproteins that help compensate for a relatively limited coding capacity. These polyproteins are processed by viral protease(s) to generate mature proteins via functional intermediates in a highly regulated manner that modulates viral replication. However, establishing how polyprotein processing regulates viral replication can be challenging due to the intricate nature of these interactions. In a previous study of the FMDV polyprotein, we showed that changing six amino acids at the 3B_3_-3C cleavage junction prevents viral replication by disrupting polyprotein processing (16). We postulated that this observation provided an opportunity to investigate the role of FMDV polyproteins in viral genome replication. First, we investigated whether consequences of substituting the six amino acids could be replicated by a single change. To do this, we substituted the P2 residue at the 3B_3_-3C cleavage junction, as our previous investigation showed this to be the most important for dictating cleavage efficiency. Using a GFP encoding replicon, we showed that a single substitution to lysine (3B_3_^T>K^) prevented viral replication and in *in vitro* translation assays changed polyprotein processing, as predicted.

To build a more complete picture of how the 3B_3_^T>K^ construct changed polyprotein processing and understand the mechanism that underpinned this change, we employed a combination of *in vitro* translation, immunoprecipitation, and *trans*-cleavage assays in the context of the FMDV nonstructural polyprotein. We found that a 3B_3_-3C cleavage junction bearing this substitution was more efficiently cleaved by 3C^pro^ in *trans* compared to WT. Furthermore, the 3B_3_^T>K^ substitution also rendered this cleavage junction sensitive to proteolysis by 3C^pro^ when the protease was present as part of a larger precursor (i.e., as part of another molecule of 3AB_1,2,3_CD). Thus, the consequence of introducing the 3B_3_^T>K^ substitution was to generate a substrate that was more efficiently cleaved (potentially equal to or greater than either 2B-2C or 2C-3A cleavage sites). This changed the subset of proteins produced; generating 2BC3AB_1,2,3_ (not typically observed during infection [7, 41]), significantly increasing levels of 3CD and 2BC and reducing levels of 3AB_1,2,3_CD. The identity of these products was based on both molecular weight and immunoprecipitation experiments. The 3B_3_^T>K^ substitution construct did generate fully cleaved 2B and 2C at a reduced rate, demonstrating that 3C^pro^ as part of 3CD is proteolytically active, but processing of 3AB_1,2,3_ and 3CD was severely impaired as observed previously (16). Further experiments to confirm the identity of these proteins could be conducted in replicon transfected cells, dependent on sufficient translation of input RNA for replication-defective replicons. It would also be interesting to investigate processing of the 3B3^T>K^ substitute when placed in the context of polyprotein containing the capsid precursor (P1-2A). This would introduce another two cleavage sites that may subtly change the hierarchy of processing of the entire polyprotein.

Our data are consistent with biochemical investigations using purified 3C^pro^ which showed that charged residues in the P2 position of the cleavage junction are more efficiently recognized (40, 42–44). They are also consistent with the suggestion that 3C^pro^ retains activity as part of a larger precursor, albeit at lower efficiency (45–48). Our data therefore agree with a mechanism of polyprotein processing (suggested from studies with other picornaviruses) in which 3C^pro^ as part of a larger precursor can process the WT 2B-2C and/or 2C-3A junctions most efficiently in *trans*, hence, providing a level of regulation to the processing cascade. After these initial cleavage events, 3AB_1,2,3_CD is processed more slowly to allow intermediate and mature cleavage products to fulfil their roles in viral replication (36). Processing of the 3AB_1,2,3_CD precursor can thus proceed potentially through both *cis* and *trans* mechanisms (i.e., intra- and intermolecular cleavage) and can give rise to alternative precursors, for example, 3AB_1,2,3_C and 3CD. It is clearly suggested from our data that *trans* cleavage of one 3AB_1,2,3_CD molecule by another polyprotein is possible. However, the WT precursor was not processed efficiently by 3AB_1,2,3_CD in *trans*, and the observation that 3C^pro^ in the context of 3CD is proteolytically active, but only able to cleave efficiently at 2B-2C and 2C-3A junctions, could suggest that other factors (e.g., a *cis* mediated mechanism), are also involved. Picornavirus 3C^pro^ proteins are also implicated in RNA binding and lipid biogenesis (potentially as part of a precursor), and therefore may act as co-factors to dictate processing pathways (29, 49, 50). A study by Escarmís et al. previously demonstrated that a single amino acid substitution in FMDV VP1 had the ability to affect a protein processing between VP1 and VP3 by 3C^pro^, despite the substitution being located 54 amino acids away from the cleavage site. This is another example of distal effects amino acid substitutions can have on proteolytic processing patterns (51).

Using *trans*-complementation assays, we investigated the function of these different sets of precursors in FMDV replication. A replicon containing the 3B_3_^T>K^ substitution (i.e., producing increased levels of 2BC, 3AB_1,2,3_, and 3CD) was able to complement defective mutations in the 3B proteins but not 2C or 3D^pol^, suggesting that this replicon can produce active primers for replication, 3B_1_, 3B_2_, and/or 3B_3_ but inactive replication enzymes, 2C and 3D^pol^. Interestingly, the 3B_3_^T>K^ replicon was only complemented by a replicon lacking *cis*-acting RNA replication elements (*cre*, S-fragment or both) and when the protein components were provided as part of an entire polyprotein. However, the PLA data suggested that the 3B_3_^T>K^ substitution does not prevent recruitment of its cognate RNA with 3D^pol^ in *trans* but instead potentially increases the association. The increased association between the 3B_3_^T>K^ RNA and 3D^pol^ provided by a WT replicon in *trans* may suggest the formation of more stable RNA-protein complexes that are detrimental to replication.

**FIG 8:**
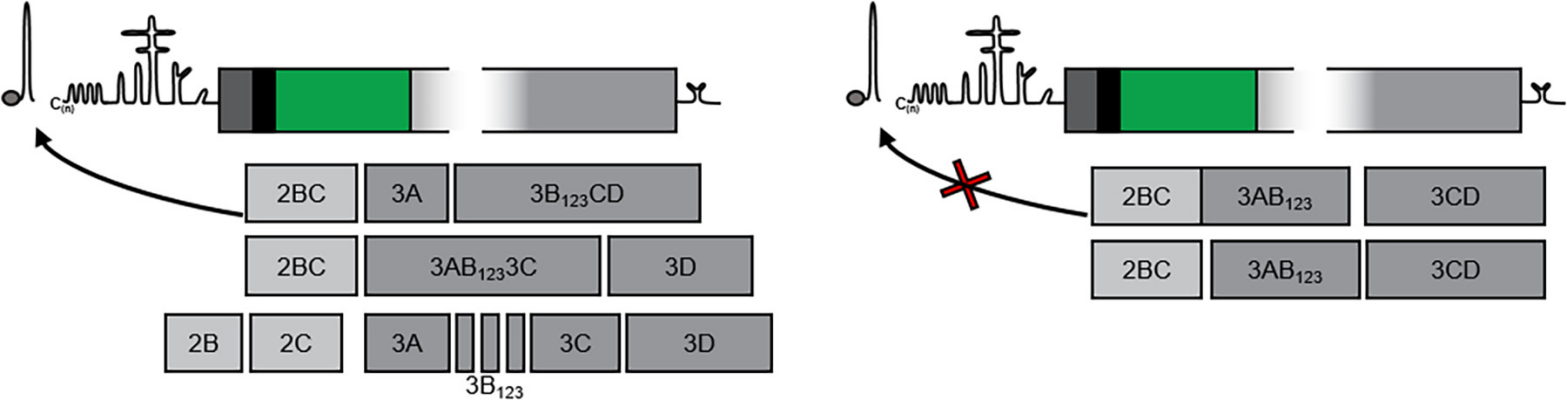
Model for regulation of FMDV replication by polyprotein processing. The 3B_3_^T>K^ substitution drives rapid processing preferentially down one pathway, as demonstrated by the reduced levels of of 2C and 3D^pol^ with increased formation of 3AB_1,2,3_ and 3CD. To support replication of the 3B_3_^T>K^ replicon in *trans*, the entire non structural polyprotein is required to deliver a complex of functional components including 2C and 3D^pol^, as demonstrated by the wild-type processing schematic. This likely provides products from alternative cleavage pathways such as 3B_1,2,3_ and 3CD.

One interpretation of our data combined is that a correct functional interaction between the nonstructural polyprotein and viral RNA elements is required to generate functional enzymes for replication (e.g., 2C and 3D^pol^ but not 3B). Hence, if processing of the precursor occurs too rapidly, it cannot associate correctly with these RNA elements required for replication, or generates a complex that is too stable (Fig. 8). These mechanisms could provide a level of temporal control of replication. This could also explain the greater number of foci seen in the PLA experiments when ptGFP-3B_3_^T>K^ BrU-labeled replicons were co-transfected with 3D^HA^ replicons (compared to co-transfections of ptGFP-3D^GNN^ or ptGFP-3B_1,2,3_^Y3F^ BrU-labeled replicon RNAs with the WT 3D^HA^ replicons). A similar mechanism has been suggested for PV where two molecules of 3ABCD are required for replication, one which produces 3CD that interacts with viral RNA structures while one produces enzymatically active 3D^pol^ (32, 36, 52, 53). This two-molecule model of processing of 3ABCD would also be compatible with data that suggest that a larger precursor (such as 3BC) is required to deliver 3B for RNA replication (15, 54, 55). This work extends the growing body of evidence suggesting that processing intermediates are essential for controlling temporally and structurally the organization of the picornavirus replication organelle.

## MATERIALS AND METHODS

### Cell lines and plasmids.

BHK-21 cells obtained from the ATCC (LGC Standard) were maintained in Dulbecco’s modified Eagle’s medium (DMEM) with glutamine (Sigma-Aldrich) supplemented with 10% FCS, 50 U/mL penicillin, and 50 μg/mL streptomycin.

Plasmids carrying wild-type FMDV replicons, pRep-mCherry and pRep-ptGFP, have already been described (38, 56), along with equivalent plasmids containing 3D^GNN^, 3B_3/2_ and ΔS-fragment mutations (16, 38, 39). Mutations within these plasmids were performed by standard two-step overlapping PCR mutagenesis. For coupled *in vitro* transcription and translation experiments pcDNA3.1(+) based expression plasmids were generated by PCR. Briefly, the relevant FMDV sequence was amplified to include flanking NotI restriction enzymes and upstream Kozak modified translational start site. The NotI digested PCR products were cloned into NotI digested pcDNA3.1(+) (Thermo Fisher Scientific). The sequence of all plasmids used in this study was confirmed by Sanger sequencing. The sequences of all primers and plasmids are available on request.

### Coupled transcription and translation reactions.

Coupled *in vitro* transcription and translation assays were performed using the TNT Quick Coupled Transcription/Translation system (Promega) as described previously (16). Reactions contained 10 μL lysate with 250 ng of pcDNA T7 expression plasmid and 0.5 μL [^35^S] methionine/cysteine (PerkinElmer). Reactions were incubated at 30°C for 40 min chasing with 2 μL of 50 mg/mL unlabeled methionine/cysteine. Reactions were stopped at 20-min or hourly intervals by the addition of 2× Laemmli buffer. Samples were separated by SDS-PAGE before visualization of radiolabeled products by autoradiography.

For the *trans*-cleavage assays, the TNT reactions were supplemented with purified FMDV 3C^pro^ (a kind gift from Dr. Tobias Tuthill [57]) to the indicated final concentration from dilution of a 1 mM stock, simultaneous to the addition of unlabeled methionine/cysteine. Reactions were stopped at 20-min or hourly intervals by the addition of 2× Laemmli buffer and the 3C^pro^-mediated proteolysis of radiolabeled precursor monitored by SDS-PAGE.

### *In vitro* transcription.

Plasmids containing cDNA copies of FMDV replicons were linearized with *Asc*I before being used to generate T7 *in vitro* transcribed RNA as previously described (38, 39). The reaction was incubated at 32°C for 4 h before being treated with DNase for 20 min at 37°C then purified using an RNA clean and concentrate kit (Zymo Research). The RNA quality was checked using a MOPS/formaldehyde agarose gel electrophoresis.

### Replication and complementation assays.

BHK-21 cells were seeded into 24-well tissue cultures vessels, allowed to adhere overnight for 16 h, before duplicate wells were transfected with 1 μg of each *in vitro* transcribed RNA using Lipofectin (Thermo Fisher Scientific) as previously described (38). For co-transfection complementation assays, 500 ng of each RNA molecules were mixed prior to the addition of Lipofectin reagent as previously described (39). Fluorescent reporter expression was monitored using an IncuCyte Zoom Dual Color FLR (Essen BioSciences) live-cell imaging system housed within a humidified incubator scanning hourly up to 24 h posttransfection. Images were captured and analyzed using the associated software for fluorescent protein expression, as previously described (39). Control transfections (untransfected and the 3D^GNN^ transfection for input translation) were used to determine fluorescent thresholds and identify positive objects from background fluorescence. A positive object was determined as having an average fluorescent intensity of >8 green calibration units (GCU; an arbitrary fluorescent unit) and >2 RCU (red calibration units), which were kept constant throughout the experiments. The number of positive cells per well was determined from the average of up to nine nonoverlapping images per well. Unless stated otherwise, data are presented as mean fluorescent positive cells per well at 8 h posttransfection when replication was approximately maximal. For each experiment, the data were analyzed as both fluorescent cell counts per well and total fluorescent intensity per well. There was no difference observed when the data were analyzed in either way. Unless otherwise stated, statistical analysis was performed using a two-tailed unpaired *t* test.

### Immunofluorescence and proximity ligation assays.

BHK-21 cells seeded onto coverslips were co-transfected with replicon RNA before fixing in 4% paraformaldehyde and washing with PBS. Immunofluorescence was conducted as previously described (39). Primary antibodies used were sheep anti-BrdU (Sigma-Aldrich), rabbit anti-FMDV 3D (a kind gift from Francisco Sobrino), and mouse anti-HA (Sigma-Aldrich). Proximity ligation assays (PLA) were conducted using the Duolink In Situ Red Kit (Sigma-Aldrich), following manufacturer’s instructions. Images from more than 20 GFP positive cells per condition were analyzed using the “Find maxima” function on ImageJ to count PLA foci across conditions with prominence set to strictly >25.

### Immunoprecipitation.

Immunoprecipitation reactions were performed using Dynabeads Protein G (Invitrogen). To bind the antibody to magnetic beads, 5 μL of the FMDV 2C antibody (a kind gift from Francisco Sobrino) was mixed with 195 μL PBS and incubated at room temperature with 50 μL magnetic beads, shaken for 1 h, after which the supernatant was removed from the beads. Transcription and translation reaction samples were mixed with 200 μL PBS and incubated shaking at room temperature with 25 μL of Dynabeads as a preclear step. The tube was placed on the magnet and the supernatant removed. This was added to the 50 μL of Dynabeads with the 2C antibody bound and incubated at room temperature shaking for 1 h. The flow through was removed and added to 2× Laemmli buffer. The beads were washed three times with PBS pH 7.4 with 0.02% Tween 20 and each wash supernatant retained. Proteins were eluted from the beads by adding 50 μL of 2× Laemmli buffer and heating to 100°C.
