# Supplementary material for: Insights into Polyprotein Processing and RNA-Protein Interactions in Foot-and-Mouth Disease Virus Genome Replication
Source: J Virol. 2023 May 8;97(5):e00171-23. doi: 10.1128/jvi.00171-23 (PMC10231256; doi:10.1128/jvi.00171-23)
Supplement: Supplemental file 1 — Fig. S1. Download jvi.00171-23-s0001.docx, DOCX file, 0.06 MB [file jvi.00171-23-s0001.docx]

**Figure S1. The 3B_3_^T>K^ substitution prevents complementation of 2C mutants *in trans*.** BHK-21 cells were co-transfected with mCherry replicons containing replication defective 2C or 3B mutations together with a WT ptGFP, ptGFP-3B_3_^T>K^ or ptGFP-3D^GNN^ replicon. Fluorescent protein expression was monitored hourly for 24 hours. The data show **(A)** ptGFP positive cells per well or **(B)** mCherry positive cells per well at 8 hours post-transfection (n = 2 ± SD).
