# Supplementary material for: Insights into Polyprotein Processing and RNA-Protein Interactions in Foot-and-Mouth Disease Virus Genome Replication
Source: J Virol. 2023 May 8;97(5):e00171-23. doi: 10.1128/jvi.00171-23 (PMC10231256; doi:10.1128/jvi.00171-23)
Supplement: Supplemental file 2 — Fig. S2. Download jvi.00171-23-s0002.docx, DOCX file, 0.06 MB [file jvi.00171-23-s0002.docx]

**Figure S2. S-fragment deletions allow *trans*-complementation of *cis-*acting replication components.** BHK-21 cells were co-transfected with mCherry replicons containing S-fragment deletions together with a WT ptGFP, ptGFP-3B_3_^T>K^ or ptGFP-3D^GNN^ replicon. Fluorescent protein expression was monitored hourly for 24 hours. The data show **(A)** ptGFP positive cells per well or **(B)** mCherry positive cells per well at 8 hours post-transfection (n = 2 ± SD).
